# FEASIBILITY AND SAFETY OF AUTOMATED MULTI-CHANNEL FES-ASSISTED GAIT TRAINING IN INCOMPLETE SPINAL CORD INJURY

**DOI:** 10.2340/jrm.v57.42638

**Published:** 2025-05-26

**Authors:** Simone BERKELMANS, Nadia DOMINICI, Maarten AFSCHRIFT, Sjoerd BRUIJN, Thomas W. J. JANSSEN

**Affiliations:** 1 Department of Human Movement Sciences, Faculty of Behavioural and Movement Sciences, Vrije Universiteit Amsterdam, Amsterdam; 2 Amsterdam Rehabilitation Research Centre | Reade, Amsterdam; 3 Amsterdam Movement Sciences, Program Rehabilitation and Development, Amsterdam; 4 Institute for Brain and Behavior Amsterdam, Vrije Universiteit Amsterdam, Amsterdam, the Netherlands

**Keywords:** functional electrical stimulation, gait-rehabilitation, incomplete, spinal cord injury, walking

## Abstract

**Objective:**

The feasibility, safety, and efficacy of automated multi-channel functional electrical stimulation-assisted gait training was assessed in individuals with chronic incomplete spinal cord injury, using an electrical stimulation suit with built-in surface electrodes and motion capture sensors (Teslasuit).

**Design:**

10-week functional electrical stimulation-assisted gait training, twice weekly for 30 min*.*

**Subjects/Patients:**

Five individuals with chronic incomplete spinal cord injury (≥ 12 months post-injury, ASIA C/D, minimal Walking Index Spinal Cord Injury II ≥ 9).

**Methods:**

The quadriceps, gluteii, hamstrings, tibialis anterior, and gastrocnemius muscles were stimulated bilaterally during gait. Feasibility and safety were evaluated via questionnaires, session adherence, and adverse events. Gait function was assessed using a 10 m walk test, Walking Index Spinal Cord Injury II, and Hoffer classification at baseline, post-intervention, and follow-up. Surface electromyography and spatiotemporal parameters (walking speed, step length and width, cadence) were recorded during the 10 m walk test.

**Results:**

All participants completed the training (91% adherence) with no serious adverse events. Temporary skin redness, muscle soreness, and fatigue were reported by participants. Post-intervention, 4 participants increased their walking speed, step length, and cadence, with 2 maintaining and 2 further improving at follow-up. No consistent changes were found in muscle activity post training.

**Conclusion:**

Automated multi-channel functional electrical stimulation-assisted gait training was feasible, safe, and well received. Preliminary findings suggest that gait improved in most participants, though individual responses varied. The results highlight the potential of multi-channel functional electrical stimulation-assisted gait training as a valuable tool for enhancing gait recovery.

## LAY ABSTRACT

In this study, we used electrical stimulation embedded in a fabric suit (Teslasuit) to activate multiple leg muscles during walking to improve walking function of people with long-term partial spinal cord injuries. Five people took part in this therapy for 10 weeks, with 2 x 30-min sessions each week. The therapy was safe and easy to follow, with high adherence to training sessions. Participants reported being able to walk faster, lift their legs more easily, and drag their feet less. Four out of 5 participants walked faster and further during the training sessions on the treadmill with stimulation. After the 10-week training, 4 participants also walked faster without stimulation compared with before the training. These preliminary results suggest that electrical stimulation during walking may help improve walking ability in people with partial spinal cord injuries. This new approach could be a valuable tool for physical therapists and rehabilitation programmes.

An incomplete spinal cord injury (iSCI) disrupts communication within the nervous system, leading to limitations in sensorimotor activities, such as walking. These limitations are associated with muscle weakness (1, 2), disturbances in reflex activity (3), and inability or difficulty in recruiting muscles below the lesion (4). People with iSCI have identified walking recovery as an important health priority, alongside bowel and bladder function (5–7). While health concerns vary depending on the level of injury, improving walking remains a key goal for those with remaining walking ability. Consequently, improving locomotor rehabilitation is of great importance.

A method that can be beneficial in this respect is using functional electrical stimulation (FES), which aims to restore functional movements by activating paralyzed or weakened muscles. Residual brain-to-spinal cord connections persist even in “clinically complete” SCI, suggesting that remaining pathways can still be influenced by external stimulation. FES can promote neuroplasticity by stimulating large-diameter afferents, activating motoneurons, and enhancing motor control through sensory feedback to supraspinal centres (8–13). Regular use of FES has been shown to result in (partial) recovery of voluntary muscle control, muscle strength, and improved gait function (14–20). FES devices have been utilized in rehabilitation of the lower extremities to improve gait parameters such as push off at pre-swing, foot clearance during the swing phase, and ground impact force at initial contact, which contribute to higher walking speeds and more effective muscle coordination during gait (17, 21, 22). Kapadia et al. (17) demonstrated that adjusting stimulation intensity and timing based on individual gait characteristics, such as matching swing phase duration to walking speed and regulating push-off force, led to signification improvements in Spinal Cord Independence Measure mobility sub-scores. Their findings suggest that individualized FES adjustments offer promising therapeutic potential for improving gait function in individuals with iSCI (17, 23).

In previous studies, the stimulation sequence was typically activated manually via a push button (17, 20, 24). However, the need to frequently press buttons presents challenges, especially for individuals with SCI who require a firm grip on support handles. Moreover, manually triggering stimulation at initial contact may also result in timing inaccuracies, causing the stimulation to occur either too early or too late, making stimulation less effective. To address these issues, we used a suit (Teslasuit, VR Electronics Ltd, London, UK) with built-in surface electrodes and inertial measurement units (IMUs) allowing automatically triggering stimulation using the IMUs that can detect initial contact in real-time.

Previous studies usually targeted only 1 or 2 lower-limb muscles with FES (24–26). Although these studies demonstrated gait improvements, it is believed that stimulating more lower-limb muscles could more effectively enhance gait patterns (20). Multiple channels can activate various movements associated with walking, such as calf muscle stimulation for push-off, hamstring stimulation for knee flexion or preventing hyperextension and glutei stimulation for hip extension and stability (27). For example, Müller et al. (28) developed an adaptive multichannel FES neuroprosthesis that generated individualized stimulation patterns, which improved foot and knee joint angles during walking, and Thrasher et al. (20) demonstrated that a multi-channel FES system, stimulating the quadriceps, hamstrings, gastrocnemius, and tibialis muscles, was effective for improving voluntary walking function. Similarly, here, we stimulated the quadriceps, glutei, hamstring, tibialis anterior, and gastrocnemius muscles. By using the Teslasuit, it is possible to deliver electrical stimulation to the leg muscles through 18 channels, consisting of shaped electrodes, distributed across 32 electrodes. This approach eliminated the need for individual electrode and IMU placements, reduces extensive wiring, and streamlines the process, making it more practical for clinical applications.

Our primary aim was to investigate the feasibility and safety of automated multi-channel FES-assisted gait training, using an electrical stimulation suit, for people with iSCI. Secondarily we assessed improvements in gait function. We hypothesized that gait function would improve after automated multi-channel FES-assisted gait training using the electrical stimulation suit. To shed some light on possible mechanisms leading to improvements in gait function, we measured leg muscle activity during overground walking before and after the intervention.

## Methods

### Participants

Participants with chronic iSCI, with the ability to independently walk 10 m overground using assistive devices and without the supervision of a physiotherapist (American Spinal Injury Association Impairment Scale Classification C or D, and a minimal Walking Index Spinal Cord Injury II of nine), were recruited (Table I). As part of the inclusion process, participants’ responsiveness to electrical stimulation was assessed using a Compex electrical stimulator (Compex SP 4.0; Compex Medical SA, Switzerland) to ensure their muscles could also be activated with the stimulation of the Teslasuit. Exclusion criteria were (*i*) cardiopulmonary disease, (*ii*) current pressure ulcers on the buttocks, (*iii*) fractures, (*iv*) unpredictable and severe autonomic dysreflexia or orthostatic hypotension, (*v*) drug or alcohol abuse, (*vi*) flaccid paralysis, (*vii*) any musculoskeletal condition that would preclude exercise, or (*viii*) participation in any FES exercise of the lower limbs during the previous 6 months. Participants were recruited by referral from clinical staff at rehabilitation centre Reade and via Neuromove, a first-line physiotherapy centre for neurorehabilitation. All participants signed written informed consent prior to participating. The medical ethical review committee of Maxima Medisch Centrum (METC number: W23.009) approved this study.

**Table I:** Participant demographic data at baseline

Participant	Sex	Age	AIS	Level of injury	Years post-injury	Most affected side	Traumatic/non-traumatic
1	Female	23	C	T11/12	2	Left	Traumatic
2	Male	47	C	C5/6	2	Right	Traumatic
3	Male	54	C	T7	5	Left	Traumatic
4	Male	46	C	T5	22	Left	Non-traumatic
5	Female	34	C	T6	4	Right	Non-traumatic

AIS: American Spinal Injury Association Impairment Score.

### FES

**Fig. 1:**
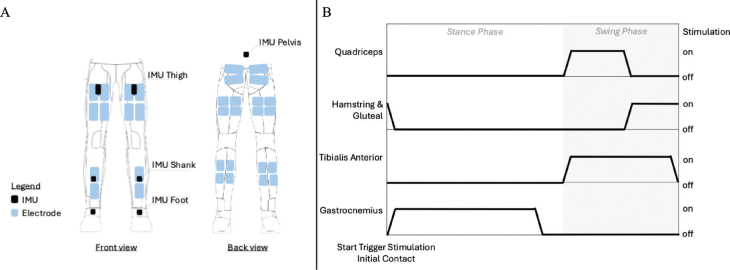
Teslasuit electrodes and experimental setup during the training. (A) Scheme of IMU and electrode placement in the Teslasuit. (B) The stimulation sequence for a single leg (left or right) initiated at initial contact of the same foot (left or right). The stimulation runs continuously, with the pre-set stimulation onset and duration, and restarts at the next initial contact of the same foot. The sequence begins and restarts at each initial contact of the stimulated leg, for both left and right.

Stimulation was applied using the Teslasuit. Participants wore the Teslasuit pants only during the training sessions. The muscle groups targeted by the FES were bilateral quadriceps, hamstrings, gluteii, tibialis anterior, and gastrocnemius muscles (Fig. 1A).

The stimulation sequence started at initial contact and assumed a constant duration of the stance and swing phase (Fig. 1B). The IMUs on the left and right foot, shank, thigh, and pelvis embedded in the Teslasuit (Teslasuit IMUs, VR Electronics Ltd, London) were used to detect gait events in real-time using a machine learning approach (https://github.com/teslasuit/walking-FES). During the first 2 weeks, adjustments to the stimulation sequence were made based on the participant’s walking speed and the duration of the stance and swing phases, as determined during baseline measurements, to optimize the walking pattern by specifically modifying the duration and timing onset of the stimulation. After this initial period, sequence adjustments became less frequent but were still made as needed. The sequence was designed as follows: during the stance phase, the gastrocnemius was stimulated to facilitate propulsion. In the subsequent swing phase, the tibialis anterior was stimulated to clear the foot from the ground. Following the initial swing, the quadriceps were stimulated to extend the knee, and during the terminal swing the hamstrings and glutei were stimulated to stabilize the leg and prevent excessive extension of the knee at initial contact.

Electrical stimulation was given at a frequency of 50 Hz, chosen to maximize muscle activation and therapeutic benefits (29–31). The pulse duration (between 1 and 320 µs) and current (~50 mA per 1 kOhm, depending on pulse duration and frequency) were determined per participant, and referred to as stimulation intensity. The stimulation intensity was set by increasing the pulse width (starting at 1 µs), during the first training session for each electrode pair and could vary among pairs. The participants were seated in their wheelchair when determining the stimulation intensity. The minimum stimulation intensity was set to the motor threshold (a visible muscle contraction occurred). The maximum stimulation intensity was set at a level that caused ± 10% of the full range of motion or became painful. The stimulation intensity was adjusted for each electrode pair throughout subsequent training sessions. Both the stimulation sequence and intensity were adjusted, seated next to the participant, based on direct visual effect on the gait pattern and participant feedback (i.e., stimulation felt too weak or too strong, too early or too late, or less effective compared with previous sessions).

### Study design

**Fig. 2:**
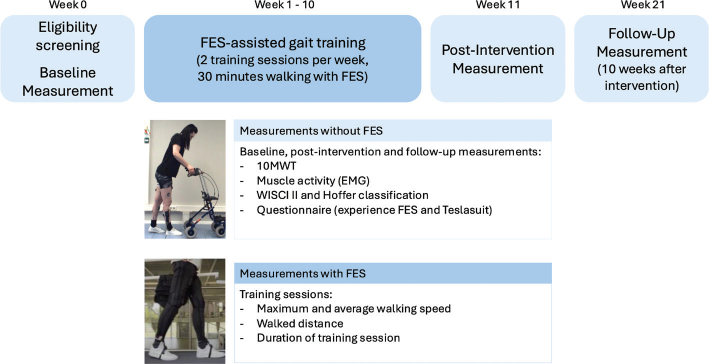
Study design. 10MWT: 10m walk test; EMG: electromyography; FES: functional electrical stimulation; WISCI II: walking index spinal cord injury II.

In the week before (baseline), after (post-intervention), and 10 weeks after (follow-up) the intervention, the Walking Index Spinal Cord Injury II (WISCI II) and Hoffer classification were determined and participants performed 3 trials of the 10-m walk test (10MWT) overground without FES (Fig. 2). Surface electromyography (EMG), videos from the front, back, and the right side, and spatiotemporal parameters were recorded during the 10MWT. During the post-intervention measurements, participants received a questionnaire for the feasibility and safety.

*Gait training with functional electrical stimulation.* Participants followed 10 weeks of FES-assisted gait training on a motorized treadmill (FDM, Zebris Medical GmbH, Isny im Allgäu, Germany, or Lode Valiant 2, Lode BV, Groningen, The Netherlands), twice a week for 30 min (excluding preparation time and breaks). The walking speed during training sessions was based on the average walking speed measured during the baseline 10MWT, with adjustments made as needed if participants struggled to maintain pace or desired a faster walking speed. All participants wore a safety harness (Guldmann GH3+ Tilmotor, Guldmann Ltd, Easingwold, UK) to prevent falls. Participants were discouraged from bearing weight through the upper extremities but could use the handrails for balance. During each session, participants could take seated breaks (with electrical stimulation paused) upon request or when researchers observed signs of fatigue.

### Data acquisition and analysis

*Feasibility and safety.* Following the post-intervention measurement, participants completed a questionnaire (Table SI). Participants were asked whether they would like to continue with FES-assisted gait training and their satisfaction with the use of FES was measured on a scale from 0 (not at all) to 10 (very satisfied). The frequency of desired training sessions per week was assessed on a scale from 1 (not at all) to 4 (5 times or more) and the preferred duration of the training sessions was measured on a scale from 1 (0 to 15 min) to 5 (longer than 60 min). Participants rated the comfort of FES while walking on a scale from 1 (very comfortable) to 5 (very uncomfortable) and provided feedback on how the FES felt during walking. Additionally, participants rated the ease of donning and doffing the Teslasuit on scales from 1 (very easy) to 5 (very difficult). Lastly, participants were asked whether the FES helped while walking on a scale from 1 (yes, definitely) to 5 (no, not at all), and to describe any positive and negative effects they experienced while using the FES during walking. Feasibility was further assessed by tracking missed training sessions and reasons for absence, instances of Teslasuit-related issues such as connectivity or stimulation timing problems, resolution of the Teslasuit-related issues, and participant dropout and reasons for discontinuation from the study. To evaluate safety, we registered any (serious) adverse events that occurred during or after the training sessions. Following each session, the researchers performed a skin inspection and asked participants about any potentially adverse events, including fatigue, muscle soreness, or discomfort. Additionally, at the start of each training session, participants were asked how they felt following the previous session and the duration of any adverse events experienced.

*10MWT and spatiotemporal gait parameters.* During each training session, the average and maximum walking speed, distance, and duration were automatically recorded by the treadmill. During the 10MWT, walking speed, step length and width, and cadence were assessed using a markerless 3D full-body motion registration system (the Interactive Walkway; https://tec4science.com/products/interactive-walkway) that uses four Microsoft Kinect v2 sensors (sampling rate 30Hz) to assess spatiotemporal gait parameters (32). Step length, step width, and cadence, calculated from the body point’s trajectories, and walking speed were determined only between the 2-m and 8-m line on the walkway to reduce the effect of gait acceleration and deceleration (33). Estimates of foot contact and foot off were required, stemming from the maxima and minima of the anterior–posterior trajectories of the ankles relative to the spine base’s trajectory (34). For the spatial gait parameters, the right and left step location were determined, defined as the median value of the right and left ankle position in the anterior-posterior and medio-lateral direction during the respective single-support stance phases (i.e., between foot off and foot contact of the contralateral foot). Based on the anterior-posterior and medio-lateral step location, step length (in cm) was calculated as the anterior-posterior difference of consecutive step locations, step width (in cm) was estimated by taking the absolute medio-lateral difference of consecutive step locations, and cadence (steps/min) was calculated from the number of steps in the time interval between the first and last estimate of foot contact.

*Muscle activity.* Surface EMG (Trigno Wireless EMG System, Delsys Inc, Natick, USA) at a sampling rate of 1000Hz was used to record activity from 7 muscles bilaterally during the 10MWTs. The muscle set comprised bilateral tibialis anterior (TA), gastrocnemius medialis (GAM), soleus (SOL), peroneus longus (PL), rectus femoris (RF), vastus lateralis (VL), and semitendinosus (ST). Participants were prepared for electrode placement by being positioned on a bed, followed by skin preparation through shaving, cleaning with isopropyl alcohol, and drying with sterile gauze. Electrode placement was guided by palpation and clinical tests, following the SENIAM recommendations (35). Trigno Avanti EMG Sensors were utilized for the upper leg muscles (RF, VL, ST), and Trigno Quattro EMG Sensors for the lower leg muscles (TA, GAM, SOL, PL). Simultaneously, a 2D clinical motion analysis system (Contemplas CMAX, Kempten, Germany) recorded the 10MWT using 3 cameras positioned at the front, back, and right side of the participant. The clinical motion analysis system was synchronized with the EMG system at the start of each trial. The 2D clinical motion analysis system was not synchronized with the Kinect system. The video recordings were used to identify the right and left foot contact events. The videos were further analysed to document the most significant gait impairments and characteristics for all participants. Muscle activity relevant to the observed gait characteristics was subsequently analysed.

Raw EMG data were processed according to the following sequence: high pass filtering at 20Hz (bidirectional fourth-order Butterworth), notch filtering at harmonics of 50 Hz (k = 1, 2, 3, 4, 5, bidirectional fourth-order Butterworth), rectification using the modulus of the analytic signal (36), and low pass filtering at 5Hz (bidirectional fourth-order Butterworth). Processed data were then segmented into strides based on foot contact and time-normalized. The EMG data were visually inspected to identify artefacts, and any corrupted data segments were excluded from further analysis. After artefact removal, EMG amplitude was normalized to the peak EMG for each muscle.

For the muscles stimulated during training, we hypothesized that training would lead to increased activity in these muscles during the periods when they were stimulated and decreased activity during periods when they were not stimulated. This would suggest more specific muscle activation in line with the stimulation pattern. To evaluate this, we calculated the average normalized muscle activity of the VL, ST, GAM, and TA during the stimulation and non-stimulation periods and computed the ratio between muscle activity during stimulated and non-stimulated periods. We then compared this parameter across assessments.

*Statistics.* Due to the small sample size and the high variability among individuals with SCI, statistical testing was not conducted.

## Results

Five individuals with chronic iSCI participated (Table II). All participants received physiotherapy before and during the study, maintaining the same weekly physiotherapy sessions. Any schedule changes were reported by the physiotherapist.

**Table II:** Participant baseline measurement outcomes

Participant	Assistive devices	WISCI II	Hoffer classification	Physiotherapy
1	Walking frame	13	Community walker	3 times per week
2	Walking sticks	16	Community walker	2 times per week
3	Walking sticks	16	Community walker	2 times per week
4	Crutches	16	Household walker	1 time per week
5	Walker, AFO	9	Therapy walker	2 times per week

AFO: ankle-foot orthoses; WISCI II: Walking Index Spinal Cord Injury III.

### Feasibility and safety

All participants completed the 10-week FES-assisted gait training programme. The percentage of completed gait training sessions was 91%, with a mean of 1.8 (SD 1.9) missed sessions per participant due to illness, other obligations, holidays, or Teslasuit-related issues. Technical issues with the Teslasuit occurred during 15 (out of 94) training sessions, including 2 instances of a broken zipper, 7 problems related to the IMUs, and 6 connection issues between the Teslasuit and the computer. All participants expressed the desire to continue FES-assisted gait training (with the Teslasuit) after the study. Satisfaction levels were high, with a median score of 7 out of 11, reflecting overall positive feedback. Participants preferred walking with FES 2–3 times per week (median score: 2/4). The preferred training duration was 30 to 45 min (median score: 3/5). Comfort during walking with FES had a median score of 3 out of 5, with the most common response being “a little uncomfortable”. Donning the Teslasuit was moderately easy (median score: 2/5) and doffing the Teslasuit was very easy (median score: 1/5). Participants reported that FES was beneficial during walking (median score: 2/5). Three participants indicated that the stimulation “helped a little bit” and 2 participants stated that it “definitely helped” during walking. Participants noted improvements such as reduced foot dragging, increased walking speed, easier movement, and better lifting of the legs when walking with FES. Moreover, participants 4 and 5 felt more supported when walking with FES. They felt that they did not need to push up on the support handles to take steps and used them only for balance (see Table SI for an overview of the questionnaire and key findings). All participants experienced temporary skin redness following each training session, which resolved within a few hours. Participants also reported experiencing muscle soreness and fatigue after training sessions, especially during the first 2 weeks of the intervention. No serious adverse events were reported.

### Gait function: spatiotemporal parameters

**Fig. 3:**
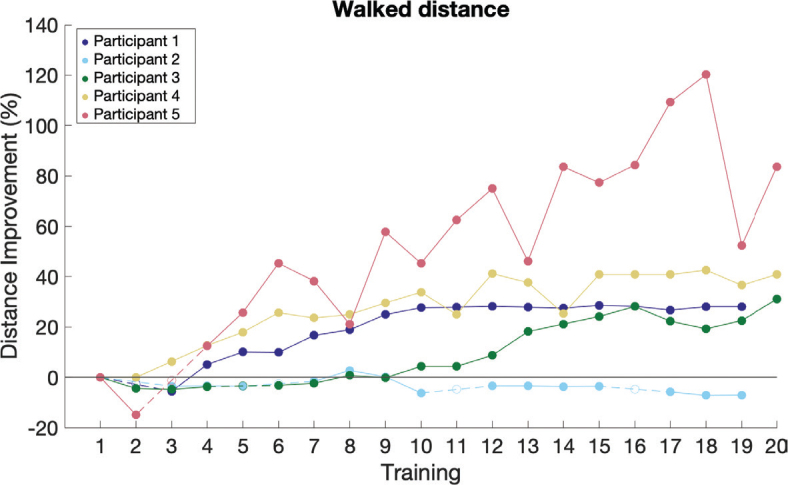
Percentage difference in walked distance during the training sessions compared with the first training session. Open circles and dotted lines indicate interpolated data for training sessions that were missed or lasted less than 30 min.

**Fig. 4:**
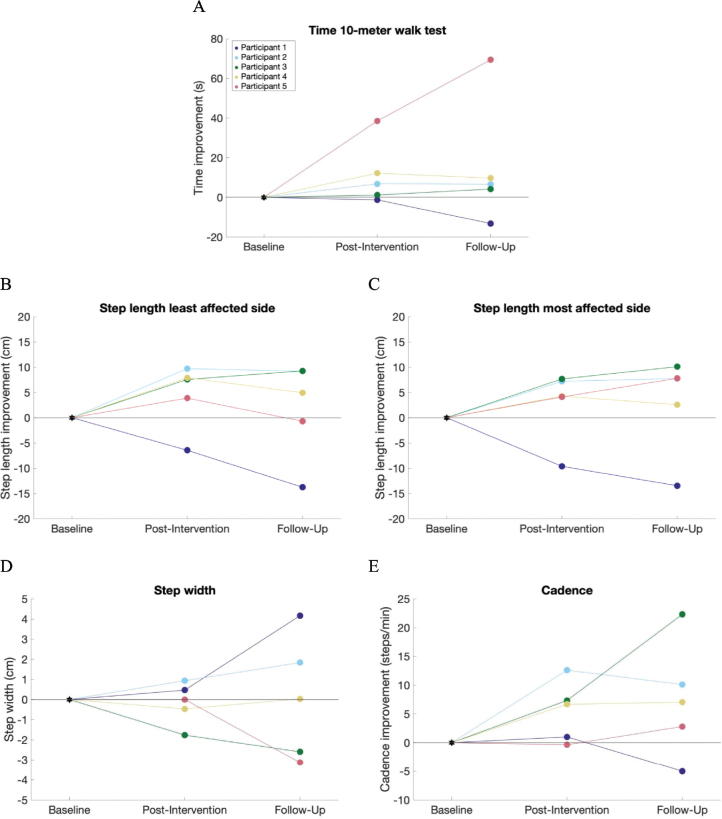
Spatiotemporal parameters during the 10MWT at post-intervention and follow-up, compared with baseline. (A) Time improvement on the 10MWT (positive indicates walking the 10MWT faster; negative indicates walking slower compared with baseline). (B and C) Difference in step length (positive indicates larger steps; negative indicates smaller steps compared with baseline). (D) The difference in step width (positive indicates larger step width; negative indicates smaller steps width compared with baseline). E) The difference in cadence (positive indicates more steps/min; negative indicates less steps/min compared with baseline).

Four of the 5 participants showed an increase in walked distance (Fig. 3), average walking speed, and maximum walking speed on the treadmill throughout the intervention. Four of the 5 participants demonstrated an increase in overground walking speed post-intervention (Fig. 4A). During follow-up, participants 2 and 4 showed a slight decrease in walking speed compared with post-intervention, but their walking speed remained increased compared with baseline. Participants 3 and 5 exhibited an increase in walking speed compared with both baseline and post-intervention. Similar trends were observed in step length, and cadence (Fig. 4B/C for step length and Fig. 4E for cadence). Step width changes were more variable, with 2 participants showing increases, 2 showing decreases, and 1 showing no change (Fig. 4D). No differences in WISCI II or Hoffer classification were observed, except for participant 5, who no longer required an ankle–foot orthosis at post-intervention and follow-up, improving their WISCI II score from 9 to 13.

### Gait function: muscle activity

**Fig. 5:**
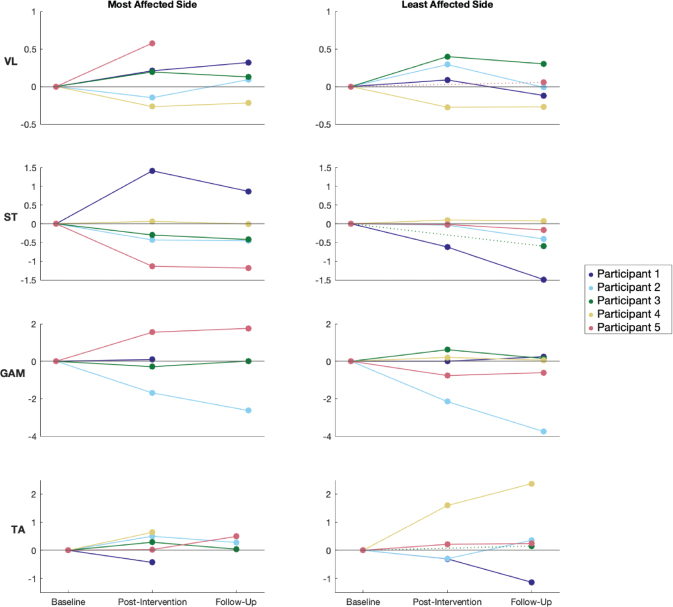
Change in stimulated/non-stimulated muscle activation ratio for VL, ST, GAM, and TA muscles between baseline, post-intervention, and follow-up. Data are shown for both the most affected and least affected sides. Each line represents an individual participant, with dotted lines indicating missing post-intervention data. VL: vastus lateralis; ST: semitendinosus; GAM: gastrocnemius medialis; TA: tibialis anterior.

EMG envelopes for all muscles and participants during baseline, post-intervention, and follow-up are shown in Figs S1–S5. Due to technical issues, a few EMG recordings for specific measurements were excluded. The ratio between the average muscle activity over the period during which the VL, ST, TA, and GAM were stimulated, and not stimulated, showed no consistent changes between baseline, post-intervention, and follow-up (Fig. 5).

## DISCUSSION

### Feasibility and safety

The primary aim of our study was to determine the feasibility and safety of automated multi-channel FES-assisted gait training for individuals with iSCI. Our findings demonstrate that automated multi-channel FES-assisted gait training is both feasible and safe. The assessment used to evaluate feasibility and safety included session completion rates, participants’ feedback, and the incidence of adverse events. All participants completed the 10-week intervention with a session completion rate exceeding 90%, which is comparable to those reported in other feasibility studies in persons with chronic iSCI (37, 38). Participants missed on average 1.8 sessions due to other obligations, illness, and technical issues. Importantly, participants indicated a strong willingness to continue this form of FES-assisted gait training. However, it is important to note that our feasibility assessment relied partly on self-reported data, which may introduce some bias or variability in responses.

The integration of electrodes and IMUs into the Teslasuit simplified the process of donning and doffing. Donning the Teslasuit took approximately 5–10 min and doffing about 2–3 min. This time efficiency might be beneficial in clinical settings, reducing preparation time. For example, in rehabilitation centres, where clients are already engaged in walking groups, the Teslasuit could be integrated into existing gait training sessions. Nurses could assist participants with donning the suit, ensuring that physiotherapists can dedicate their available time to walking with FES rather than preparation. Despite the overall positive outcomes of using the Teslasuit to apply electrical stimulation, technical issues were encountered during 15 (out of 94) training sessions. In 12 sessions, the problems were completely or partially resolved, allowing participants to continue walking with the correct stimulation sequence. However, in 3 sessions, unresolved issues (a broken zipper and a connection problem) resulted in either shortened sessions or sessions without FES. Consequently, these sessions were classified as missed training sessions in our feasibility analysis.

A proper fit of the Teslasuit is crucial for effective stimulation. A well-fitted suit ensures that the electrodes and IMUs are correctly positioned on the body, which is essential for delivering electrical stimulation to the targeted muscles. If, however, the suit is too large, the electrodes may not make proper contact with the skin, leading to ineffective or absent muscle stimulation or high voltages, resulting in more skin redness and pain. In our study, we had 2 different suit sizes available, but it will be essential for future research and clinical practice to provide a broader range of suit sizes to accommodate different body types.

The safety of the FES-assisted gait training was also evaluated. All participants experienced temporary skin redness following each training session, which resolved within a few hours. Participants also reported muscle soreness and fatigue after training sessions, especially during the first 2 weeks of the intervention. The temporary skin redness and muscle soreness are common and expected side effects of FES, and they did not deter participants from continuing the training. No serious adverse events were reported, indicating that the training is safe for individuals with iSCI.

### Improvements in gait function

The secondary aim of our study was to evaluate whether FES-assisted gait training could improve gait function after the intervention and during follow-up. Four out of 5 participants demonstrated improvements in overground walking speed, step length, and cadence post-intervention. While 2 participants showed a slight decline at follow-up compared with post-intervention, their gait function remained improved compared with baseline. These findings align with previous studies (17, 20, 24, 39–41), indicating that individuals with chronic iSCI can experience gait and mobility improvements through functional training, even years after injury. Notably, 2 participants continued to improve at follow-up, potentially due to increased motivation and walking frequency post-intervention. Our findings underscore the importance of continued rehabilitation in chronic iSCI, even 12 months post-injury, when further improvements might not typically be expected.

Variability in response to the FES-assisted gait training was observed among participants, with some participants showing more substantial improvements than others. Factors such as lesion level, injury severity, and individual response to FES may contribute to this variability. For instance, participant 1 did not show improvements in overground walking speed, step length, and cadence post-intervention and experienced a decline at follow-up. This unexpected outcome contradicts the expectation that gait function would remain stable due to the chronic nature of the participants’ lesions. A plausible explanation could be fatigue, as the participant walked to the laboratory before the 10MWT at follow-up, unlike at baseline and post-intervention when a wheelchair was used. Another possible explanation is that participant 1 might have denervation signs in the stimulated muscle due to the level of the lesion, which could contribute to the variability in response. However, at baseline, the Compex device indicated that all muscles were stimulable, suggesting functional lower motor neurons capable of responding to FES. Despite the lack of spatiotemporal improvements, participant 1 showed notable improvements in gait pattern during training with FES and at post-intervention without FES (see Fig. S1).

Four out of 5 participants increased their average walking speed and distance during training sessions, aligning with previous findings (20). However, participant 2 did not show progress during training sessions, potentially due to the highest number of missed sessions among all participants. Despite this, participant 2 exhibited improvements during the 10MWT in spatiotemporal parameters, underscoring the complexity of individual responses to FES.

### Gait pattern

During the examination of participants’ walking patterns, several common difficulties were identified, including difficulty lifting their feet during the swing phase, limited clearance, reduced push-off, and knee overextension during both the landing and swing phases. There was also a noticeable reliance on assistive devices such as crutches or walkers, with participants often needing to push up on these devices before taking steps. After the intervention, the recorded videos suggest improvements in overground walking patterns, such as a better ability to lift their feet during the swing phase, leading to improved clearance, and a reduction in knee overextension. These gait improvements may be explained by neural plasticity and motor control reorganization. Residual brain-to-spinal cord connections persist even in “clinically complete” SCI, suggesting that remaining pathways can still be influenced by external stimulation (8, 42). Neural networks in the spinal cord are critical for posture and locomotion (9–11), and FES can promote neuroplastic changes. By stimulating large-diameter afferents, FES activates alpha-motoneurons and engages antagonistic muscles through polysynaptic interneuron connections (12, 13). Additionally, proprioceptive afferents relay sensory feedback to supraspinal centres, enhancing motor control through neuroplasticity (13). For instance, participant 1 showed excessive hip flexion, characterized by kicking the feet forward, at baseline. The kicking motion became less pronounced during training sessions and post-intervention, which aligns with EMG data from the RF and VL muscles (see Fig. S1), which showed 3 bursts of activation (kicking and overextension) at baseline but only 1 burst (less pronounced kicking) post-intervention. Additionally, Participants 1, 4, and 5 demonstrated better ability to lift their feet during the swing phase post-intervention.

### FES customization

To evaluate the effects of the FES sequence on muscle activity, we analysed the average muscle activity of the VL, ST, TA, and GAM. We hypothesized that, even when not walking with FES anymore, FES-assisted gait training would increase muscle activity during stimulated periods and reduce activity during non-stimulated periods, potentially explaining the observed gait improvements. However, the data revealed considerable inter-individual variability in muscle activation ratios, with no consistent trends across participants or muscles, indicating that the impact of the intervention is highly individualized (see Fig. 5). This variability is also reflected in the EMG patterns for participants 1 to 5 (see Figs S1–S5).

The stimulation sequence and intensity were customized for each participant based on the individual’s response. Although perception of stimulation intensity varied across sessions – possibly due to factors such as the SCI, muscle soreness from previous sessions, or differences in suit fit – the ability to adjust stimulation intensity during each session, and for each electrode pair, allowed for a level of personalization that likely contributed to the overall effectiveness of the intervention. Moreover, the detection of initial contact via the IMUs made applying the FES sequence during gait more convenient for researchers and physiotherapists compared with the push-button method, allowing the researchers to focus on the participants’ gait patterns and adjust the stimulation sequence and intensity as needed. For the participants, it kept their hands free to hold onto the support handles for balance.

The stimulation sequence was fine-tuned to optimize timing and duration of the stimulation during the gait cycle. Based on visual inspection and participant feedback, adjustments were made to the stimulation sequence by modifying the onset time and duration of the stimulation. Additionally, adjustments were made to the stimulation intensity during each training session for each electrode pair. The stimulation frequency was set at 50 Hz, which may be too high, as such higher frequencies have been shown to lead to faster onset of muscle fatigue (30). There are, however, many variables that affect the optimal settings, and it is not clear what the best level is for each individual participant. To mitigate fatigue, stimulation intensity was carefully adjusted for each electrode pair based on participant feedback and visual inspection, with adjustments made throughout the sessions to account for fatigue. Training sessions were paused when participants showed signs of fatigue, and FES was temporarily halted.

Simplifying the process of adjusting the stimulation sequence and intensity would be beneficial for future studies. In this study, the FES sequence was triggered by the detection of initial contact only. By detecting additional gait phases, we could more accurately determine the timing of gait events and estimate the duration of the full stride, as well as both the stance and swing phases. With this information, the stimulation sequence could be adjusted automatically, rather than manually, thereby enhancing both the performance of the FES system and the overall user experience.

### Limitations

Several limitations should be considered when interpreting the results of this study. The small sample size limits the generalizability of our findings, and the absence of a control group precludes definitive conclusions regarding the efficacy of FES-assisted gait training compared with gait training without FES or other conventional rehabilitation approaches. Future studies with larger sample sizes and randomized controlled designs are necessary to further investigate the effects of automated multi-channel FES-assisted gait training on gait function in individuals with SCI.

Correct stimulation timing (see Fig. 1) in our study relied on the detection of initial contact, participant feedback, and observational data from side-view analyses during training sessions. However, we cannot confirm with certainty that the stimulation occurred at the intended times. Incorporating a mechanism to record stimulation output would provide insight into the alignment between intended and actual stimulation.

### Conclusions

Our results show that automated multi-channel FES-assisted gait training is both feasible and safe for individuals with iSCI. The high session completion rate and positive participant feedback underscore the practicality of this intervention. While technical issues were encountered, they were generally manageable, and the integration of the Teslasuit streamlined the process, making it suitable for clinical settings. Participants experienced common, temporary adverse effects such as skin redness and muscle soreness. Additionally, we found that most participants showed improvements in gait. These improvements persisted to some extent at follow-up. Future advancements in automated adjustment of the stimulation sequence, to individualize for each participant, could further enhance the user experience and performance of the FES system. Despite the small sample size and lack of a control group, our findings provide valuable insights into the potential of FES-assisted gait training. Future studies with larger sample sizes and randomized controlled designs are needed to confirm these results and further explore the efficacy of this intervention.

## Supplementary Material

Supplementary material has been published as submitted. It has not been copyedited, or typeset by Journal of Rehabilitation Medicine

Supplementary material has been published as submitted. It has not been copyedited, or typeset byJournal of Rehabilitation Medicine

## Trial details

NCT05908175..
